# Development and validation of a broadly applicable instrument to measure patients’ health promotion and empowerment process in chronic disease

**DOI:** 10.1177/17423953241306268

**Published:** 2024-12-10

**Authors:** Kristin Heggdal, Kisha Thompson, Natalie Stepanian, Krystyna de Jacq, Keville Frederickson

**Affiliations:** 1 Faculty of Health Sciences, 87368 VID Specialized University, Oslo, Norway; 2 College of Health Professions, 116470 Pace University, Lienhard School of Nursing, Pleasantville, USA

**Keywords:** Chronic illness, health promotion, person-centered, scale development, scale validity

## Abstract

**Objectives:**

This study aimed to develop and validate a generic, non-disease-specific, self-assessment measure that recognizes patients’ health capacities and their empowering process of health promotion in chronic illness by using Bodyknowledging as the theoretical frame.

**Methods:**

Item generation and expert content validity analysis were the first steps in instrument development. Potential items were then validated in focus group interviews with six patients diagnosed with various chronic diseases. The research team reviewed the resulting items and undertook item reduction. A sample of adults (n = 357) with chronic disease surveyed the instrument items. Exploratory factor analysis with Oblimin rotation was conducted.

**Results:**

A 3-factor solution was identified: nine items on the *regaining health and wellness* subscale, seven items on the *uncertainty and bereavement* subscale, and eight items on the *loss of control and distancing* subscale. Cronbach alpha for the scale was .92. The final 24-item instrument is non-disease-specific and broadly applicable for use in health promotion within the context of chronic disease. The instrument demonstrates high internal consistency reliability with initial face and construct validity.

**Discussion:**

The new instrument has the potential for measuring patients’ empowering process of health promotion in chronic disease and the outcomes of person-centered interventions and may guide clinicians in tailoring individual support.

## Introduction

There is an urgent need for fundamental change in society's attitudes towards people with chronic disease: Emphasis should be placed on their potential for health, not on disease and deficit.^1,2^ This change is needed to facilitate empowerment and to ensure that all available resources are utilized to handle the worldwide epidemic of chronic disease and increased pressures on healthcare systems.^3^ Chronic diseases such as chronic obstructive pulmonary disease (COPD), cardiovascular diseases, diabetes, cancer, and mental illnesses are characterized as non-communicable disease (NCDs) and have become the dominating group of illnesses in the world.^4^ These illnesses are followed by reduced quality of life and shortened life expectancy, especially in low or middle-low economic societies. Sustainable Development Goals have set the target of reducing premature mortality from NCDs to one-third by 2030 by means of prevention, treatment, and health promotion.^5^ Individual, community, and national entities are challenged to join efforts to attain these goals.^2^

Research findings indicate that people who are diagnosed with chronic disease possess resources and strategies for self-management and health that are not fully recognized and, therefore, sub-optimally utilized.^6–8^ However, emphasis on empowerment and health promotion is vital for engaging patients in their healthcare to improve clinical outcomes and quality of life.^9,10^ In recent decades, the chronic disease management paradigm has shifted its emphasis from “being cared for” towards active patient participation. The implication is that patients are expected to take an active role in preventing secondary illnesses and promoting their health.^11^ However, the bridge between engaging patients with chronic disease in health promotion during clinical practice and utilizing their health capacities must be constructed.

### Health promotion and empowerment in chronic disease

Health promotion is defined as enabling people to increase control over and to improve their health.^12^ It has traditionally been associated with preventive measures for healthy people, while for those diagnosed with chronic disease, health promotion has been poorly described in the literature. One of the few sources found is Huckstad, who describes health promotion in chronic disease as involving “behavioral change for positive lifestyle activities, accepting one's condition and making the necessary adjustments, decreasing the risk of secondary disabilities, preventing further disease, and striving for optimal health”.^13^ The definition emphasizes behavioral change as the first step in health promotion. The earlier writings of Kaplun^1^ are more in line with the World Health Organization's (WHO) definition of health promotion, as she asserts that

Health promotion in chronic disease is a process of enabling and developing potentials for healing and health which implies developing new strategies and actions to strengthen hope among sufferers, to reduce their anxieties and to facilitate a meaningful life. Its goal is to increase the capacity of people to deal with the consequences of chronic illness and to ensure that this experience does not dictate their lifestyle. (413)

Furthermore, Kaplun^1^ invites the person and their family to explore ways of dealing with everyday stresses and anxieties that do not necessarily require medical intervention, pointing instead to the value of supportive environments and personal skills that will maximize healing—“a process emerging from the inner resources of the individual” (412). The question, then, becomes: How can a patient's health promotion process emerging from the person's inner resources for health be facilitated to increase control over and improve their health while living with chronic disease? In alignment with this goal, the construct of interest and focus of this paper is how patients’ empowering process of health promotion in chronic disease can be measured by means of a new generic instrument to be used across diagnostic categories.

The concepts of patient participation, patient empowerment, and partnership between patients and healthcare professionals have been introduced as an attainable goal for strengthening patient health and wellness in chronic disease.^14,15^ Patient participation, empowerment, and health promotion are closely linked as patient participation requires the person to have power in order to execute prevention and health promotion individually and in collaboration with healthcare professionals.^16,17^

Empowerment has been described as a concept, a process, and an outcome. A wide variety of definitions exists in the literature with no unified description. For example, Mora et al.^18^ reviewed 49 studies where 35 different descriptions were used to define empowerment. The existing definitions included patients’ capacity, power, knowledge, activities or behaviors, shared decision-making, and management of illness and lives. Furthermore, concepts such as enablement, engagement, activation, and personal control were identified as related to empowerment and highlighted patients’ important role in prevention and health promotion work while living with chronic disease.^19^

The lack of a clear conceptualization of empowerment as well as the interchangeable use of empowerment with other concepts suggest the need to distinguish between empowerment and health promotion as a process, and self-management, self-efficacy, patient activation, health literacy, behavioral change, and quality of life as indicators or outcomes of patient empowerment. This study was based on the understanding of empowerment as an integrated part of the person's health promotion process in chronic disease, including the four fundamental components of the empowerment process as defined by WHO^2,20^: patient participation, patient knowledge, patient skills, and the creation of a facilitating environment.

### Measures of health-related empowerment in chronic disease

Several systematic reviews were conducted to attain an overview of measures used to assess patient empowerment in chronic disease. Mora et al.'s^18^ review identified 38 different instruments that were applied to measure empowerment. Two other reviews of health-related empowerment measurements mainly focused on parents or family members, children, community empowerment, or sociopolitical control, including disease- and situation-specific instruments.^21,22^ A fourth review was aimed at cancer patients.^23^ In addition, two reviews were conducted to identify health-related empowerment measures to be used across diagnostic categories. Barr et al.'s^24^ review included all types of patients, with 30 studies reporting 19 different measures. No clear consensus was reached across studies on patient empowerment as a driving force for health, and a wide variety of patient-reported outcomes was used. Most measurements were disease-specific, and only six of 19 instruments were described as generic. Two of the six generic measures included empowerment only on the subscale level, one evaluated empowerment in drug therapy, and one focused on hospital staff's actions to empower patients. The Health Education Impact Questionnaire (HeiQ) developed by Osborne et al.^25^ and the Health Care Empowerment Inventory (HCEI) developed by Johnson et al.^26^ were also generic assessments.

In Pekonen et al.'s^19^ systematic review, 13 instruments were identified as generic; nine of them were developed for empowerment in different long-term conditions for adults or older people. These nine instruments were: HeiQ, HCEI, Patient Perception of Empowerment (PPES), Small et al.'s instrument, a Chinese version of Client Empowerment Scale (CCES), Patient Enablement Instrument, Patient Health Engagement Scale, an instrument for Hong Kong's Special Administrative Region, and Multidimensional Health Locus of Control Scale. The structure of these instruments was heterogeneous; the number of subscales ranged from two to eight, while six instruments did not have any subscales. The number of items ranged from five to 47. Responses were measured on 3-point to 6-point Likert scales, with the 5-point used most frequently (strongly disagree-strongly agree). The content of the instruments was variable. All 13 measurements identified as generic instruments in Pekonen et al.'s^19^ review included the elements of patient capacity and patient knowledge, and most included patient behavior; however, only five instruments included all four fundamental elements of empowerment defined by WHO.^2,20^ These were the HeiQ, PPES, CCES, Health Empowerment Scale (HES), and Patient Activation Measurement (PAM).

To summarize, Barr et al.^24^ identified the HeiQ and HCEI as generic instruments to be used across diagnostic categories. Pekonen et al.^19^ identified the HES, CCES, and PAM as generic instruments to measure empowerment most comprehensively and with acceptable evidence of validity and reliability. When looking more closely at these instruments, their focus was on important elements of health-related empowerment in chronic disease. However, we have yet to identify a generic instrument that measures patient empowerment and health promotion as a process.

This study aimed to develop and validate a generic, non-disease-specific, self-assessment measure that recognizes patients’ inherent health capacities and the empowering process of health promotion in chronic disease to be used to evaluate the outcome of the interventions.

## Methods

A four-phase instrument development and validation study was conducted between May 2019 and April 2023. This study was approved by Pace University IRB [1731270–2] and follows the World Medical Association Declaration of Helsinki. The study was conducted according to procedure, and the reporting of results was based on best practices for developing and validating scales for health, as described by Boateng et al..^27^

### Phase 1: defining the theoretical framework for the new instrument

Bodyknowledging theory^28,29^ served as the main concept and theoretical framework for the development of the new instrument because the theory describes the patients’ process of health promotion and empowerment in chronic disease across diagnostic categories. The theory asserts that individuals living with long-term health problems possess bodily knowledge that constitutes an important inherent resource for empowerment, self-management, health, and wellness that is developed through a dynamic process interacting with the environment. Bodyknowledging is defined as “a fundamental process for the development of personal knowledge about one's own body, coping skills, health and wellbeing”.^28^ The process comprises the physiological, psychosocial, and existential dimensions of the person living with chronic disease. It involves the patient's embodied knowledge, that is, the knowledge of self, one's body, and individual experiences that is developed over several overlapping and non-linear phases: Uncertainty—denying and escaping the sick body; Losing life space—grieving and anger; Listening to and learning the body`s signs and signals—strengthening hope; and Integrating knowledge—new possibilities for wellness and health.^28,29^

Bodyknowledging theory was developed using grounded theory and drew on several studies involving in-depth interviews from patients diagnosed with different long-term conditions, such as stroke, COPD, inflammatory bowel disease (IBD), and a variety of other diagnoses. Patients’ experiences of their illness and health, coping abilities, and health promotion resources were central to this work. Empirical findings demonstrated two major concerns of people with chronic disease: the multitudes of uncertainties they face and the restrictions of life space (the sphere to act within one's daily life) encountered due to their health condition.^28^ Patients’ accounts of embodied knowledge of health and illness stood out as a powerful tool for managing their life with chronic illness. It became a helpful resource through the Bodyknowledging process including movement in alternating phases of learning and health-related change. The phases are dynamic and overlapping, and the person's work on the different phases is challenging. The work involves moving on a health-promoting continuum that does not have a set starting or ending point; rather, it is more of a health-promoting direction in which to move. While this theory aligns with the theory of salutogensis,^30^ it also adds to this theory by eliciting the importance of the person's bodily knowledge as a powerful, inherent resistant resource that creates movement towards the healthy end of the continuum. The process involves integrating different types of experience and expertise in a personal knowledge base by which one can experience health within illness and improvement even when living with a chronic disease.^28^

Bodyknowledging theory served as a frame for developing a new health promotion intervention named The Bodyknowledging Program (BKP).^29,31^ The BKP intervention has been tested and found to be broadly applicable across diagnostic categories and clinical settings.^29,32,33^ Against this background, we aimed to develop a new and generic instrument to use across diagnostic categories and clinical settings to measure the outcome of the BKP intervention and possibly other health-related empowerment interventions in chronic illness focusing on the activation of the persons’ inherent resources for health.

### Phase 2: scale development

An international collaboration team of four researchers was formed to design the instrument. The theory expert, a senior professor and expert in qualitative methodology, a co-Investigator trained in research methodology, and a co-Investigator trained in quantitative methods initiated the study and worked closely on scale development. Furthermore, an expert panel for the content validity phase included two additional faculty familiar with the theoretical framework and two healthcare practitioners with extensive experience employing Bodyknowledging theory in clinical practice in Norway.

#### Item generation

All team members reviewed the theory by reading published and unpublished literature.^28^ One hundred ten items were initially generated representing the four conceptual phases of Bodyknowledging theory. Each item contained a single concept at the sixth-grade reading level and was measurable by a Likert scale. The team's theoretical and qualitative experts reviewed the items to fit with Bodyknowledging theory. They indicated that some aspects of the theory were not incorporated and suggested additional items, resulting in 130 items. These items were grouped by theoretical phases. The team considered interpretability, ambiguity, positive and negative wording, length of items, double-barreled statements, value-laden words, and high cognitive load before reducing the number of items from 130 to 99.^34^

#### Expert content validity

The 99 items were reviewed for face and content validity to determine if they were relevant to the content being measured. Two Norwegian experts and two American interdisciplinary scientific collaborators familiar with the theory reviewed the items to obtain the experts’ judgement on how well the items reflected Bodyknowledging theory. They rated each item on a scale of 1 to 4 for clarity of statement and relevance to related concept. The team reviewed the expert ratings and retained all items that received a rating of 3 or 4 without modification; this resulted in 61 items kept for further testing through focus group interviews.

### Phase 3: focus group validation of scale

A focus group for evaluation and validity testing was held in September 2021. Participants were recruited from a community of independently living senior citizens in suburban New York. Eligibility criteria included: (1) self-management of at least one chronic disease (i.e., multiple sclerosis, epilepsy, Parkinson's, heart failure, stroke, cancer, COPD, severe chronic pain, lupus, arthritis, diabetes, chronic IBD) and demonstration of cognitive capacity by answering open-ended eligibility criteria questions; (2) having been ill for more than 1 year preceding the study and having health problems that in some way limited their health status, everyday activities, or daily functioning. Participants of any gender and ethnic background were eligible to participate as long as they could speak and understand English. Exclusion criteria were (1) having mental illnesses, and (2) lacking decisional capacity. Before the focus group, an email was sent to participants including an article to familiarize them with Bodyknowledging theory.^28^

Six participants participated in the focus group; all were women diagnosed with chronic diseases, including congestive heart failure, COPD, asthma, spinal stenosis, cardiac valve repair via open heart surgery, atrial fibrillation requiring frequent hospitalizations, severe rheumatoid arthritis, and chronic Lyme disease (ongoing Borrelia burgdorferi infection). Ages ranged between 70–79. All had obtained a minimum education of a bachelor's degree and were self-managing their chronic disease.

Focus groups allowed for the evaluation and validation of the items. Participants were asked to review items on the current draft of the instrument for clarity and relevance (Appendix 1. Interview Questions). The qualitative expert and another co-Investigator were co-facilitators of the focus group. Participants reviewed the consent form and agreed to be videotaped, giving their verbal consent on the recording for participation, videotaping, and confidentiality. The proposed process of evaluating 61 items for a potential new instrument was reviewed for over 2 h; during this time, the focus group examined each item, and also considered the items collectively.

Participants made recommendations, which were noted in writing for the group to visualize, and each revision was again reviewed with the participants. Where there was disagreement about a change, revisions continued until all participants reached consensus. Gift cards for $10 were emailed to participants when the focus group was completed.

Forty-two items had suggested revisions, while seven new items were introduced. The participants noted that the order of item presentation should go from more compromised to higher levels of Bodyknowledging theory (i.e., begin with items in the uncertainty and bereavement phase and progress toward the regaining health and wellness phase). After the focus group, the co-facilitators reviewed the recommended changes and confirmed the findings related to each question. Results from the focus group were presented to the theory expert for first impressions; then, the research team collectively reviewed each item. Consensus was reached on item wording and placement, with consideration of input from the focus group. These steps resulted in a draft instrument with 66 items suitable for a pilot study. Several examples of the focus group's influence on item revisions are presented in Table 1.

**Table 1.** Examples of focus group influence on item revisions.

Bodyknowledging Theory Phase	Presented Item	Focus Group Feedback	Revised Item
Uncertainty – denying and escaping the sick body	I want to escape whatever this is that is making me feel bad	Comment: Too similar to following item	Removed
	I hope the illness will disappear	Comment: Use in place of previous item	I hope the illness will disappear
	I’m losing control of my life	Comment: How relevant is this to theory?	Ìm losing control of my body`s reactions
Losing life space – grieving and anger	I am worried about everything I cannot do	I am anxious/sad about some of the things I cannot do.	I am sad about some of the things I cannot do.
	Suffering takes away time for work, fun, and a social life.	My illness takes away time for work, fun, and a social life.	My illness takes away time from work.
Listening and learning the body`s signs and signals – strengthening hope	My own body provides me with specific knowledge.	Unchanged	Unchanged
	I can listen to my body to manage my illness.	Comment: Better fit in this phase	Moved to this phase, wording unchanged
Integrating knowledge – new possibilities for wellness and health	I have reconciled with my health situation	I have accepted my health situation	I have accepted my health situation
	I know how to prevent relapses	I know how to deal with setbacks	I know how to prevent setbacks
	New recommendation:	Diet, exercise, sleep habits demonstrate better ability to manage chronic illness.	In order to improve my health, I do physical exercise.

### Phase 4: survey administration

Data collection and analysis for exploratory factor analysis (EFA) were conducted in 2022–2023. The Bodyknowledging Questionnaire (BKQ) items were entered into Qualtrics, and a Uniform Resource Locator (URL) link was created and placed on the recruitment flyers along with a Quick Response Code that would direct participants to the survey. The inclusion criteria were as follows: English speaking, 18 years or older, having had a chronic disease for over a year, and facing health-related problems.

The first recruitment approach was purposive sampling engaging volunteer undergraduate senior nursing students in a community health course to distribute flyers to potential participants during their clinical experiences at low-income independent senior high-rises within the New York City metropolitan area and Westchester County, NY, yielding n = 8. Next, researchers distributed recruitment flyers through snowball sampling through Facebook, garnering n = 30. Then, senior nursing students were asked to distribute flyers through Facebook. Students received a $5.00 Amazon gift card for each completed survey, contributing n = 6. Next, administrators and directors of online chronic disease support groups were contacted to post recruitment flyers on their Facebook sites. Initially, this strategy obtained n = 16 completed surveys, and the process was slow. After 4 weeks, $15.00 Amazon gift cards were offered as incentives for participants, resulting in a steady flow of surveys over the next 3 months. Amazon gift cards were emailed to those who successfully completed the survey.

## Results

### Sample

The respondents’ (n = 357) mean age was 32.1, with 157 males (43.9%), 122 females (34.1%), 3 participants identifying as other, and 76 nonresponses (21.1%). Participants self-reported their racial identity as being White (234, 65%), African American (30, 8.4%), or another race (18, 5.1%). Most respondents were college-educated (129, 36%) and had full-time employment (208, 58.1%). Participants reported the following chronic diseases: arthritis (54), cancer (13), chronic IBD (56), COPD (21), diabetes mellitus (34), epilepsy (10), heart failure (13), lupus (8), multiple sclerosis (7), Parkinson's (2), and severe chronic pain (23); most attested to having more than one chronic disease (123). All completed surveys were retained and analyzed using SPSS version 28.0. We purposefully targeted recruitment efforts toward online chronic disease organizations and support groups to minimize the risk of random engagement by those who did not meet eligibility criteria.

### Item reduction

Inter-item correlations between 66 items were examined. Items that were over- or under-correlated items were removed (the threshold range was .3–.7) resulting in a 61-item instrument. The number of items was further reduced using expert judgment to reduce the redundancy of the items. We asked the experts to review the items again for alignment with the theoretical model. The experts worked independently on the assessment and reached a consensus. Thirty-eight items were selected for further testing.

### Extraction of factors

The EFA for content validity^35^ allowed for an examination of underlying dimensions to explain the relationships between the items and eliminate redundant questions by reviewing factor loadings (Table 2). We performed an EFA using Oblimin rotation, assuming that the items were correlated with the Bodyknowledging theory. We retained items with loadings above a threshold of .3 on each factor and removed 14 items that cross-loaded on two factors, obtaining a final solution with 24 items. The Kaiser-Meyer-Olkin test was .93, well above the minimum acceptable value of .60, indicating that the data set was appropriate for factor analysis. Bartlett's sphericity test was significant (X^2^(276) = 32934.2; *p* < .001), indicating that the variables in the population correlation matrix were correlated. The number of factors extracted was decided using an eigenvalue threshold of > 1. EFA identified three principal factors that explained 53.2% total variance. Cronbach alpha coefficient for the scale was = .92. Factor 1, labeled the *regaining health and wellness* subscale, consisted of nine items (α = .89); factor 2, labeled the *uncertainty and bereavement* subscale, consisted of seven items (α= .83); and factor 3, labeled the *loss of control and distancing* subscale, consisted of eight items (α= .86). The scale demonstrated high internal consistency reliability with initial face and construct validity.

**Table 2.** Factor loadings.

Items	Factor 1 Regaining health and wellness	Factor 2 Uncertainty and bereavement	Factor 3 Loss of control and distancing
I no longer feel secure.		.780	
I am losing control of my body's reactions.		.833	
I hope the illness will disappear.			−.854
I don’t want to be sick.			−.731
My illness is unpredictable.		.649	
I am sad about some of the things I cannot do.			−.504
The body rules my life.		.690	
I wonder why this happened to me.			−.494
I think about how my life will evolve with this illness.		.406	
I cannot manage everything as before.		.671	
My illness takes away time from work.		.658	
I can listen to my body to manage my illness.	.795		
My own body provides me with specific knowledge.	.498		
I can take charge of my health.	.767		
I am learning how to manage my ongoing health.			−.585
I search for new opportunities for wellness.			−.602
I have accepted my health situation.	.751		
Understanding my health allows me to move on.	.584		
I am able to be healthy despite having a chronic condition.	.732		
I know what support I need from others.			−.566
Knowing my limits will enable me to do more.	.556		
I know how to prevent setbacks.	.643		
I hope my health will get better in the future.			−.780
In order to improve my health, I do physical exercise.	.647		

## Discussion

### Summary of the main findings

This study aimed to develop and validate a non-disease-specific, self-assessment measure that recognizes patients’ intrinsic empowering process of health promotion in the face of chronic disease. The instrument was based on Bodyknowledging theory^28^ and describes phases or stages of a person's empowering process of health promotion, which involves learning awareness about the body's signals, understanding how the signals can be interpreted, and determining how symptoms and discomfort can be managed.

Bodyknowledging is specifically about learning what contributes to health, wellness, and recovery while living with illness; it works to enable people to increase control over and improve their health.^29,36^ A variety of diagnostic categories was represented in the development and testing of the theory, which makes it relevant as a foundation for the development of a non-disease-specific, generic instrument to measure patients’ empowerment process of health promotion in chronic disease.^29^

The BKQ consists of 24 item and three subscales. The factor 1 subscale captures how the person is *regaining health and wellness* and growing confidence in their capability for managing health; that is, it demonstrates the possibilities for experiencing health within illness. These responses indicate that the person has worked through phases of Bodyknowledging and developed expert knowledge and skills, by which the person has strengthened and established new possibilities for health and wellness. The factor 2 subscale, *uncertainty and bereavement,* represents feelings of grief and doubt. These responses suggest that the person recognizes the impact of the disease but is overwhelmed by the loss of previous health and frustrated by limitations. The factor 3 subscale exhibits negative factor loadings and is characterized by notions of *loss of control and distancing* through avoidance and attempts to escape the chronic illness. At the same time, although there may not be evidence of traditional coping mechanisms, there is a fight to believe in healing and hope for wellness. These three factors capture the phases described in the theory of Bodyknowledging,^2,28,29^ albeit not in the same way as in the theory. Factor 1 *regaining health and wellness* incorporates two phases of Bodyknowledging: Listening and understanding the body's signs and signals—strengthening hope; and Integrating knowledge—new possibilities for wellness and health. Factor 2 *uncertainty and bereavement* captures the phase of Losing life space—grieving and anger. Factor 3 captures the phase of Uncertainty—denying and escaping the sick body. Factor 1 indicates a high level of empowerment and health, factor 2 indicates a lower level, and factor 3 indicates the lowest level of Bodyknowledging—hence, a low level of empowerment and health. Thus, the theoretical phases are blended in the resultant factors, which is to be expected because the theory emphasizes that the phases are not distinct but overlapping in that one phase may shade into another. The theory recognizes the unique, non-linear process where people living with chronic disease move in and out of phases in their empowering process of health promotion.^28,29^

### Comparison to existing literature

According to WHO,^20^ there are four fundamental components of the empowerment process to attain health promotion in chronic disease: patient participation, patient knowledge, patient skills, and creation of a facilitating environment. We argue that patients’ inherent process of Bodyknowledging and their bodily knowledge of health, illness, and skills developed through this process constitute an essential core of empowerment and health in chronic disease and is reflected in the new instrument.

Former studies confirm that patients’ experience-based knowledge constitutes a critical but underutilized resource for preventing deterioration and building self-management and health in chronic disease.^37,38^ Patients’ bodily knowledge of chronic illness is multidimensional, consisting of personal knowledge of one's limits and tolerances of the type and amount of activity; physical and psychosocial factors in the patients’ environment that positively or negatively impact their condition; and personal knowledge of symptoms of relapses as well as the actions, interactions, and social contexts that contribute to recovery in their specific life situations.^28,29^ These capacities align with the first three fundamental factors of empowerment: patient participation, patient knowledge and patient skills.^20^ The fourth factor, the creation of a facilitating environment to strengthen empowerment and health in chronic disease, was identified as social context characterized by knowledge, understanding, and hope.^29^

Existing instruments capture essential elements of health-related empowerment and outcomes. The new instrument is a novel contribution because it is a measure of *patients’ empowering process of health promotion as they perceive it*, which is an important aspect not previously measured in existing instruments. Furthermore, existing instruments measure the outcome or engagement with health professionals who work to empower patients, which seems to reflect a received view—namely, the view of empowerment as something given to patients from health care professionals. Thus, by contrast, BKQ measures empowerment and health as patients’ own process of health promotion in which they activate themselves.

Patient participation and development of personal knowledge, skills, and capacity to handle chronic disease and promote health is described in the theory of Bodyknowledging and reflected in the subscales of the BKQ instrument. This adds a person-focused dimension to measuring health and empowerment, as compared to other generic measurements like PAM, HeiQ, HES, HCEI, and CCES.^19,24^ Furthermore, the new instrument adds the dimension of process to the measurements of patient empowerment and health in chronic disease and elicits the BKQ as a new option for evaluating the outcome of person-centered interventions.

The questionnaire development and validation methodology resulted from a thorough examination of global literature and best practices.^27,39^ The resultant measure underwent quantitative EFA to establish three factors in a predominantly online sample of American adults with self-reported chronic disease and demonstrated that the BKQ has good validity and reliability.

#### Strengths and limitations

The (BKQ) instrument can be applied across a wide range of chronic conditions and settings because the concepts of the theory are grounded in patients’ experience of health and illness across diagnoses and clinical sites. Using lay concepts as tools of an instrument is an advantage to ensure that the instrument is built on patient participation and, by this, is perceived as relevant for the target population. A thorough qualitative methodological process was conducted to generate items, explore content validity and test relevance, lead to item reduction and explorative factor analysis, and result in a 24-item questionnaire which is easy to use in a variety of practices. In addition to representing the patient's perspective, the process element of a person's empowerment and health promotion as captured in the BKQ is a major strength and adds substantially to existing instruments.

Some limitations should be considered when interpreting the results of this study. The first language of the content experts was a mix of Norwegian and American English speakers. The focus group was demographically specific which can be regarded as a weakness, however, the item generation of the scale rests on Bodyknowledging theory which was grounded on the basis of in-depth interviews with patients representing a variety of demographic characteristics. Severe mental illness was exclusion criteria for the focus group due to the fact that the theory was not grounded in this diagnostic population. Including people with severe mental illness would be an interesting future project.

A large portion of the Phase 4 sample was from online sources with self-reported eligibility. According to Boateng et al.,^27^ Step 3 in developing scales involved scale evaluation by means of (a) tests of dimensionality to test if latent constructs are as hypothesized; (b) tests of reliability to establish if responses are consistent when repeated; and (c) further tests of validity to ensure that the instrument measures the latent dimensions intended. Step 3 was omitted because we lacked resources and faced other obstacles during the COVID pandemic.

### Implications for future research and clinical practice

Engaging patients with chronic disease in health promotion and utilizing their health capacities are mandatory for future clinical practice to enhance the longevity, quality of life, and health of millions of people who are diagnosed with chronic disease and to reach Sustainable Development Goals.^5^

Results from clinical trials demonstrated how the Bodyknowledging framework can be used as a frame for an intervention to sustain and facilitate empowerment and health in encounters with people facing chronic disease.^29^ The results are promising, as measured by qualitative interviews, the Antonovsky's Sense of Coherence Questionnaire,^40^ and the Outcome Rating Scale.^33^ However, a specific instrument like the BKQ is needed, and will advance the assessment of the outcome of the BKP intervention in order to guide health professionals in how they tailor their support in practice. The concepts of Bodyknowledging theory were used to develop subscales that allow clinicians to assess “where the patient is” in their process and what their needs are in terms of support to regain wellness. Therefore, the BKQ is specially designed for measuring the outcome of the BKP intervention. Future research will demonstrate if the instrument can also serve as a non-disease-specific, generic instrument to measure the outcome of other interventions and will entail Step 3 in scale evaluation^27^ as well as confirmatory analysis.

This study also demonstrated one process of using a grounded theory based on patient experiences to develop an instrument and may inspire others to utilize theory on patient capacities for health in the development of future scales.

## Conclusion

This study resulted in the development and validation of a broadly applicable instrument that captures patients’ empowering process of health promotion in chronic disease. Content and face validity were strong and aligned with the four fundamental components of empowerment as defined by WHO. The BKQ instrument can be applied across a wide range of chronic conditions and settings because the theory grounding it reflects patients’ empowering process of health promotion across a variety of diagnostic categories.

## Supplemental Material

sj-docx-1-chi-10.1177_17423953241306268 - Supplemental material for Development and validation of a broadly applicable instrument to measure patients’ health promotion and empowerment process in chronic disease Supplemental material, sj-docx-1-chi-10.1177_17423953241306268 for Development and validation of a broadly applicable instrument to measure patients’ health promotion and empowerment process in chronic disease by Kristin Heggdal, Kisha Thompson, Natalie Stepanian, Krystyna de Jacq and Keville Frederickson in Chronic Illness

## References

[1] Kaplun A. Health promotion and chronic illness. Discovering a new quality of health. Copenhagen: WHO Regional Office for Europe, 1992. 1514968

[2] World Health Organization. Voice, agency, empowerment: Handbook on social participation for universal health coverage, 2021.

[3] Anderson G Rega ML Casasanta D , et al. The association between patient activation and healthcare resources utilization: a systematic review and meta-analysis. Public Health 2022; 210: 134–141. 35970015 10.1016/j.puhe.2022.06.021

[4] World Health Organization. Global status report on noncommunicable diseases. Geneva: WHO, 2011.

[5] Bennett J Stevens G Mathers C , et al. NCD Countdown 2030: worldwide trends in non-communicable disease mortality and progress towards Sustainable Development Goal target 3.4. Lancet 2018; 392: 1072–1088. 30264707 10.1016/S0140-6736(18)31992-5

[6] Kristjansdottir OB Stenberg U Mirkovic J , et al. Personal strengths reported by people with chronic illness: a qualitative study. Health Expect 2018; 21: 787–795. 29478260 10.1111/hex.12674 PMC6117496

[7] Paterson B . The myth of empowerment in chronic illness. J Adv Nurs 2001; 34: 574–581. 11380725 10.1046/j.1365-2648.2001.01786.x

[8] Carel H Kidd IJ . Epistemic injustice in healthcare: a philosophical analysis. Med Health Care Phil 2014; 17: 529–540. 10.1007/s11019-014-9560-2 24740808

[9] Graffigna G Barello S Bonanomi A , et al. Measuring patient engagement: development and psychometric properties of the Patient Health Engagement (PHE) Scale. Front Psychol 2015; 6: 247. 25870566 10.3389/fpsyg.2015.00274 PMC4376060

[10] Hibbard J Greene J . What the evidence shows about patient activation: better health outcomes and care experiences; fewer data on costs. Health Aff (Millwood) 2013; 32: 207–214. 23381511 10.1377/hlthaff.2012.1061

[11] Haugan G Eriksson M . Health promotion in health care - vital theories and research. New York: Springer, 2021. 36315659

[12] World Health Organization. The Ottawa Charter for Health Promotion, Ottawa 1986, https://www.who.int/publications/i/item/WH-1987 (1987).

[13] Larsen PD . Lubkin’s chronic illness. Impact and intervention. 10th ed. Burlington, MA: Jones & Bartlett Learning, 2019.

[14] Palumbo R Annarumma C Manna R , et al. Improving quality by involving patient. The role of health literacy in influencing patients’ behaviors. Int J Healthc Manag 2021; 14: 144–152.

[15] Hickmann E Richter P Schlieter H . All together now – patient engagement, patient empowerment, and associated terms in personal healthcare. BMC Health Serv Res 2022; 22: 1–11. 36056354 10.1186/s12913-022-08501-5 PMC9440506

[16] Naidoo J Wills J . Foundations for health promotion practice. 4th ed. London: Elsevier, 2016.

[17] Freire P . Pedagogy of the oppressed. 50th anniversary edition ed. New York: The Continuum, 2017.

[18] Mora MA Sparud-Lundin C Moons P , et al. Defintions, instruments and correlates of patient empowerment: a descriptive review. Patient Educ Couns 2022; 105: 346–355. 34140196 10.1016/j.pec.2021.06.014

[19] Pekonen A Eloranta S Stolt M , et al. Measuring patient empowerment: a systematic review. Patient Educ Couns 2020; 103: 777–787. 31767243 10.1016/j.pec.2019.10.019

[20] World Health Organization. Patient empowerment and health care. *WHO Guidelines on Hand Hygiene in Health Care: First Global Patient Safety Challenge Clean Care Is Safer Care*. World Health Organization, 2009. 23805438

[21] Herbert RJ Gagnon AG Rennick JE , et al. A systematic review of questionnaires measuring health-related empowerment. Res Theory Nurs Pract 2009; 23: 107–132. 19558027 10.1891/1541-6577.23.2.107

[22] Cyril S Smith BJ Renzaho AM . Systematic review of empowerment measures in health promotion. Health Promot Int 2016; 31: 809–826. 26137970 10.1093/heapro/dav059

[23] Eskildsen NB Joergensen CR Thomsen TG , et al. Patient empowerment: a systematic review of questionnaires measuring empowerment in cancer patients. Acta Oncol (Madr) 2017; 56: 156–165. 10.1080/0284186X.2016.1267402 28077053

[24] Barr PJ Scholl I Bravo P , et al. Assessment of Patient Empowerment: a systematic review of measures. PLoS One 2015; 10: 1–24. 10.1371/journal.pone.0126553 PMC4430483 25970618

[25] Osborne RH Elsworth GR Whitfield K . The Health Education Impact Questionnaire (HeiQ): an outcomes and evaluation measure for patient education and self-management interventions for people with chronic conditions. Patient Educ Couns 2007; 66: 192–201. 17320338 10.1016/j.pec.2006.12.002

[26] Johnson MO Rose CD Dilworth SE , et al. Advances in the conceptualization and measurement of health care empowerment: development and validation of the Health Care Empowerment Inventory. PLoS One 2012; 7: e45692. 10.1371/journal.pone.0045692 PMC3446922 23029184

[27] Boateng GO Neilands TB Frongillo EA , et al. Best practice for developing and validating scales for health, social and beavioural research: a primer. Front Public Health 2018; 6: 1–18. 29942800 10.3389/fpubh.2018.00149 PMC6004510

[28] Heggdal K . Utilizing bodily knowledge in patients with chronic illness in the promotion of their health: a grounded theory study. Calif J Health Promot 2013; 11: 62–73.

[29] Heggdal K . Health promotion among individuals facing chronic illness - The unique contribution of The Bodyknowledging Program. In: Haugan G Eriksson M (eds) Health promotion in health care – vital theories and research. New York: Springer Publisher (in press), 2021, pp.209–226. 36315719

[30] Antonovsky A . Unraveling the mystery of health: how people manage stress and stay well. San Fransisco, CA: Jossey-Bass, 1987.

[31] Heggdal K . ‘We experienced a lack of tools for strengthening coping and health in encounters with patients with chronic illness’: bridging theory and practice through formative research. Int Pract Dev J 2015; 5: 1–16.

[32] Heggdal K . Patient's experience of the outcomes of engaging in a broadly applicable health promotion intervention for individuals facing chronic illness. Health (NY) 2015; 7: 765–775.

[33] Heggdal K Oftedal B Hofoss D . The effect of a person-centred and strength-based health intervention on recovery among people with chronic illness. Eur J Person-Cent Healthc 2018; 6: 279–285.

[34] Streiner D Norman G Carney J . Health Measurement Scales: a practical guide to their development and use. New York: Oxford University Press InC, 2015.

[35] Tavakol M Wetzel A . Factor analysis: a means for theory and instrument development in support of construct validity. Int J Med Educ 2020; 11: 245–247. 33170146 10.5116/ijme.5f96.0f4a PMC7883798

[36] WHO. The Ottawa Charter for Health Promotion, https://www.who.int/teams/health-promotion/enhanced-wellbeing/first-global-conference (1986).

[37] Wilde MH . Embodied knowledge in chronic illness and injury. Nurs Inq 2003; 10: 170–176. 12940971 10.1046/j.1440-1800.2003.00178.x

[38] Thorne SE Ternulf Nyhlin K Paterson BL . Attitudes toward patient expertise in chronic illness. Int J Nurs Stud 2000; 37: 303–311. 10760537 10.1016/s0020-7489(00)00007-9

[39] Ricci L Lanfranchi J Lemetayer F , et al. Qualitative methods used to generate questionnaire items: a systematic review. Qual Health Res 2019; 29: 149–156. 29952223 10.1177/1049732318783186

[40] Heggdal K Lovaas B . Health promotion in specialist and community care: how a broadly applicable health promotion intervention influences patient`s sense of coherence. Scand J Caring Sci 2018; 32: 690–697. 28699654 10.1111/scs.12498

