# Supplementary material for: Development and validation of a broadly applicable instrument to measure patients’ health promotion and empowerment process in chronic disease
Source: Chronic Illn. 2024 Dec 10;21(3):361–74. doi: 10.1177/17423953241306268 (PMC12405642; doi:10.1177/17423953241306268)
Supplement: sj-docx-1-chi-10.1177_17423953241306268 - Supplemental material for Development and validation of a broadly applicable instrument to measure patients’ health promotion and empowerment process in chronic disease [file sj-docx-1-chi-10.1177_17423953241306268.docx]

Appendix 1. Interview Questions

The participants were asked:

- What do you think about this item?
- Does this item reflect what you have experienced living with chronic illness?
- Is the item easy to understand?
- Is the item difficult to answer?
- Does the item reflect the related phase of Bodyknowledging theory?
